# Exploring the use of musculoskeletal ultrasound imaging by podiatrists: an international survey

**DOI:** 10.1186/s13047-021-00478-4

**Published:** 2021-05-12

**Authors:** Charlotte Dando, Richard Ellis, Matthew Carroll, Prue Molyneux, Gabriel Gijon-Nogueron, Heidi J. Siddle, Lindsey Cherry, Alfred Gatt, Catherine Bowen

**Affiliations:** 1 School of Health Sciences, Faculty of Environmental and Life Sciences, University of Southampton, Highfield Campus Building 67, University Road, Southampton, Hampshire, SO17 1BJ UK; 2 School of Clinical Sciences, Auckland University of Technology, Auckland, NZ New Zealand; 3 Active Living and Rehabilitation: Aotearoa New Zealand, Health and Rehabilitation Research Institute, School of Clinical Sciences, Auckland University of Technology, Auckland, NZ New Zealand; 4 Department of Nursing and Podiatry, University of Malaga, UMA, Málaga, Spain; 5 Leeds Institute of Rheumatic & Musculoskeletal Medicine, University of Leeds, Leeds, UK; 6 The Academy of Research and Improvement, Solent NHS Trust, Southampton, UK; 7 Faculty of Health Sciences, University of Malta, Msida, Malta; 8 Centre for Sport, Exercise and Osteoarthritis Research Versus Arthritis, Southampton, UK

**Keywords:** Ultrasound imaging, Podiatry, Impact, Musculoskeletal

## Abstract

**Background:**

Podiatrists, in musculoskeletal services, are demonstrating an expansion of their practice skills through the use of ultrasound imaging. There is an assumption that this practice is beneficial within the context of patient care and health systems. The aim of this research was to further investigate the use of musculoskeletal ultrasound (MSUS) by podiatrists within their clinical setting and gain additional insights into the impact that they perceive use of MSUS has on their approaches to management of musculoskeletal foot and ankle problems.

**Method:**

An international study utilising a cross-sectional design and an internet-based platform was undertaken. The survey was developed and implemented through three phases: 1. survey development, 2. face validity agreement via questionnaire review, and 3. survey distribution and data collection. Twenty-two survey questions were developed and set as a two-step approach collecting quantitative data (part 1) and qualitative free text data (part 2). Data was exported from SurveyMonkey and analysed using Microsoft Excel software. Counts and frequencies were calculated for responses to all twenty closed questions. Responses to the two final open-ended questions were analysed using thematic analysis to search for patterns related to podiatrists’ perceptions of impact.

**Results:**

Two hundred and thirty-two eligible participants consented to complete the survey. The majority (*n* = 159) of respondents were from the UK and Spain. Commonly MSUS has been used in practice for (i) diagnosing pathology, (ii) supporting rehabilitation, (iii) supporting interventions or (iv) research purposes. Most frequently, MSUS was used to assist in the diagnosis of injury/pathology (84%). A range of free text comments were received from the participants in response to the question relating to their thoughts on the impact of using MSUS imaging in their practice (*n* = 109) and on their perceptions of how the use of MSUS has influenced their approaches to management of their patients’ musculoskeletal foot and ankle problems (*n* = 108). Thematic analysis of the free text comments generated four themes: (i) diagnosis, (ii) delivery and access of care, (iii) patient education and engagement, and (iv) patient empowerment.

**Conclusion:**

The perceived benefit podiatrists indicated in using MSUS as part of their practice is the perceived improvement in patient journeys through tighter, focused management plans and reduced waiting times. An additional novel finding was that MSUS provided the capacity for podiatrists to better inform patients of their diagnosis, which they believed led to improved engagement and consequent empowerment of patients in their treatment plans. We propose further investigation of patient experiences as well as testing of the model that embeds podiatrists’ use of MSUS as a key skill in musculoskeletal foot and ankle services.

**Supplementary Information:**

The online version contains supplementary material available at 10.1186/s13047-021-00478-4.

## Background

The use of ultrasound imaging to examine parts of the body was first reported in 1958 [1]. Professions such as radiology, rheumatology and cardiology were the first to describe and report on the application of ultrasound imaging in supporting their clinical decision making [2–7]. Musculoskeletal ultrasound (MSUS) can reliably quantify some bone and most soft tissue changes with exceptions such as bone oedema and fractures [5, 6]. MSUS provides the ability for real-time, dynamic and multiplanar assessment of joint pathology, allowing the examiner complete control to fully investigate the area of interest [8]. Consequently, the use of MSUS at the point of care facilitates efficiencies in clinical decision making and evidence of improvement in patient outcomes and improvements in care [8].

The increasing use of MSUS has progressed to Allied Health Professionals [9]. Two recent surveys have reported on the increasing use of ultrasound by physiotherapists [10] and podiatrists [11]. Both surveys confirmed an increase in use of MSUS by physiotherapists and podiatrists and at the same time emphased the importance of training competencies and recommendations from professional bodies to promote safe practice. The international survey of physiotherapists’ use of MSUS in their practice, revealed that over a third (38%, *n* = 497) of physiotherapists (*n* = 1307 responders) were using MSUS in their clinical practice [10]. The uses of MSUS, offered by respondents to the survey, were broadly grouped into six areas: biofeedback; diagnosis; assessment; injection guidance; research and teaching [10]. The survey of podiatrists using ultrasound imaging in their clinical practice was confined to UK podiatrists (*n* = 294 respondents) and included vascular hand-held Doppler imaging, therapeutic ultrasound applications, and use of diagnostic MSUS [11]. Thirty seven percent (*n* = 105) of respondent podiatrists reported regularly using diagnostic MSUS in their practice. Recommendations from both surveys focussed on the development of competency frameworks to underpin training and clinical governance [10, 11]. In the case of both inquiries, there was a common assumption that the use of MSUS is beneficial within the context of patient care and health systems.

The expansion of expertise in musculoskeletal services of podiatrists using MSUS supports the UK health policy agenda, where demographic changes have driven workforce redesign, which features the promotion and expansion of flexible working [12–15]. As it stands, there is limited published evidence in other countries using MSUS to draw upon, hence why there is a UK bias. Anecdotally, neither New Zealand nor Australia offer a formal MSUS accreditation for podiatrists thus imbedding into practice is slower. Consequently, accurately reporting perceived impact to patients and health systems is challenging. Therefore, further highlighting the importance of identifying the evidence gaps where future international collaboration is needed.

To effect a revolutionary change for growth in use of MSUS within podiatry further research is required to understand the context in which MSUS is used by podiatrists in their clinical setting in different health systems and how their use of MSUS impacts on their approaches to management of their patient’s musculoskeletal foot and ankle problems. The aim of this research was to further investigate the use of musculoskeletal ultrasound (MSUS) by podiatrists within their clinical setting and gain additional insights into the impact that they perceive use of MSUS has on their approaches to management of musculoskeletal foot and ankle problems.’

## Methods

A two-step approach using a cross-sectional survey design was chosen to identify how podiatrists were using MSUS within their clinical setting (1. quantitative multiple-choice response questions) and to gain insights into the impact that they perceived use of MSUS has on their approaches to management of their patients’ musculoskeletal foot and ankle problems (2. qualitative free text response questions). In order to understand the context of different health systems, this survey used an international internet-based platform undertaken to facilitate international reach.

Participants included consenting, registered podiatrists. The survey was developed and implemented through three phases: 1. survey development, 2. face validity agreement via questionnaire review, and 3. survey distribution and data collection. This study was carried out in full accordance with the Declaration of Helsinki on ethical principles and was approved by the University of Southampton Ethics and Research Governance Committee in December 2019 (Reference number: 54018).

### Survey development

This survey was adapted from two previously undertaken by Ellis et al. [10] and Siddle et al. [11] that examined the use of ultrasound imaging within physiotherapy and podiatry, specifically the scope of practice and the type and content of ultrasound imaging training. Previous investigations had focused on the broader application of ultrasound. This survey focused on MSUS and understanding the clinical use of MSUS by podiatrists and the impact that podiatrists think MSUS has on patient care. (See supplement file 1).

### Survey agreement

Twenty-two survey questions were developed, collecting a mixture of quantitative and qualitative data. This included 20 multiple choice questions with pre-selected phrases or values for participants to select. Additional comment boxes were made available if further detail was relevant. The two final questions were open-ended giving participants the freedom to write their thoughts and opinions in their own words on the perceived impact MSUS has on patient care and services (see supplement file 2). All questions were reviewed by a collaborative group of MSUS experts to check face validity until agreement was achieved. Face validity was determined by the extent to which the questions were subjectively viewed as covering the concept it proposed to measure for example, MSUS used in clinical practice and perceived potential impact. The experts were identified through their academic and clinical contributions to MSUS of the lower limb. Our experts were those identified as authors of this paper. They were invited to review the study aims and objectives, the questions’ intent and meaning, the flow of the survey as well as the logic of following the online processes. The collaborative group consisted of podiatrists (n = eight: UK, Malta, Spain, New Zealand) and one physiotherapist (New Zealand) who all currently use MSUS for clinical, teaching or research purposes.

### Survey distribution and data collection process

The survey was hosted on the internet-based survey site, SurveyMonkey that enabled secure anonymous survey participation. Although anonymised, the SurveyMonkey report did reveal the IP addresses of respondents. The way we set up our surveys meant that SurveyMonkey would not allow multiple answers from the same IP address, so multiple responses were not possible. The SurveyMonkey site created a web-based link that could be used on social media platforms. When participants clicked on the survey link, they were taken to a description of the study purpose, evidence of ethical approval, and the participant information sheet. Informed consent was obtained via the participant ticking the ‘next’ button to continue onto the survey questions.

A purposeful sampling strategy was employed via posting of survey links on social media pages of special interest groups and sending emails to known podiatrists using MSUS. The authors had a combined network of twitter followers to be approximately 4400. The approximation of international podiatry specialist groups in sonography, on social media platforms, was approximately 8800 followers. There was an appreciation that not all individuals who are a part of the specialist groups wound be suitable to take part in the survey. A snowball technique was then encouraged for participants to share and forward the link through media posts, email or group meetings. The virtual advertising of the survey was repeated monthly for three months.

### Participants

The target audience were podiatrists who were currently using MSUS within their practice. It was estimated that our survey sample would be 1200 podiatrists. This figure was an approximation from review of international podiatry specialist groups in sonography. The inclusion criteria required podiatrists who were registered with a national licensing organisation/registration authority and were using MSUS. Podiatrists were invited to self-identify whether they would be eligible to take part. The survey took approximately 10 min to complete. The submitted surveys were anonymous, with no personal identifiers, responses could not be linked back to the individual. The SurveyMonkey online platform was password protected.

### Data analysis

Data were exported from SurveyMonkey (Copyright© 1999–2020 SurveyMonkey) and analysed using Microsoft Excel software (Microsoft 365®, Microsoft Corporation, 2018. Microsoft Excel). Counts and frequencies were calculated for responses to all 20 closed questions. Each question was analysed individually, with data reported as per number of participants who answered this question as the online platform for data collection could allow participants to skip questions. Responses to the two final open-ended questions were analysed using thematic analysis to search for patterns related to podiatrists’ views. Codes were generated by noting recurring comments that were then used to categorise responses by two researchers (CD and CB). Using constant comparison, the codes were refined, compared and grouped into similar features which served as potential themes (CD and CB). Potential themes were repeatedly discussed by CD and CB to identify any alternative interpretations. The process of verifying themes with the wider team (RE, MC, PM, GGN, HS, LC, AG) provided a rigorous approach, incorporated different perspectives and led to the agreement on the final themes. (see supplement file 3).

## Results

### Survey response

Two hundred and thirty two eligible participants who consented to complete the survey. For each question, the Internet-based survey site was able to indicate how many participants completed the question and how many skipped the question. This is highlighted specifically for each of the findings. The completion rate for the survey was 1 (232/232) and the completeness rate was 0.46 (108/232) (see supplement file 4).

### Professional and demographic characteristics

Podiatrists who completed the survey practiced as a podiatrist or as a podiatric surgeon in the following countries: United Kingdom (86), Spain (73), Canada (29), The Netherlands (27), Australia (8), Malta (3), Italy (2), Ireland (1), Kenya (1) and South Africa (1). It was not unusual for participants to report a mixed employment profile, and this was reflected in the responses. The majority of podiatrists or podiatric surgeons indicated their type of work was clinical (208; 92%) followed by teaching/education (36; 16%), research (29; 13%), management 12; 5%) and other (2; 0.8% but not stated what this included). Five participants skipped this question.

**Fig. 1:**
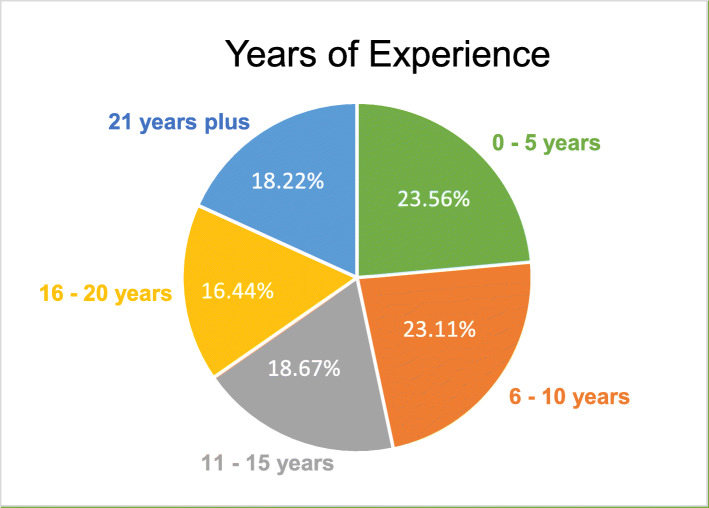
Years of experience reported by Podiatrists

As was seen for employment, it was not unusual for participants to report a mixture of clinical practice settings, with those indicated: private practice (153; 67%), public hospitals/clinic (71; 31%), academic institutions (49; 22%), private hospital (26; 11%), private organisation (18; 8%), research facility (6; 3%), sports teams (5; 2%), community (charity, support groups) (5; 2%), unrelated to the clinical field of podiatry (marketing or sales) (1; 0.4%) and other (e.g. workforce management 2; 0.8%). Participants reported a varied skill mix of assessing and managing conditions associated with the lower limb. The term ‘other’ included orthopaedics, haemophilia, anaesthetics and cosmetic education. Participants (*n* = 225) also reported a range of 0–25 years’ experience worked in practice (Fig. 1).

### Use of MSUS by podiatrists in the clinical setting

Responses (*n* = 135) indicated that participants were commonly using MSUS imaging for (i) diagnosis of injury/soft tissue pathology, (ii) injection and needle guidance, (iii) to monitor healing and treatment outcomes and (iv) research. Most frequently, MSUS was used to assist in the diagnosis of injury/pathology (84%), for measuring linear soft tissue (e.g. muscles, tendons, nerves) thickness and width (76%), for guiding percutaneous procedures (e.g. acupuncture, dry needling, needle guidance etc.) (69%), as an indicator for Doppler activity (67%), and for evaluating muscle structure (e.g. shape, muscle fascicle length, fatty infiltration etc.) (60%). MSUS used for the assessment of soft tissue trauma (59%), monitoring healing (59%), and the monitoring of treatment outcomes (e.g. from comparing a baseline parameter to the same parameter at follow-up) (57%) was less frequent.

Participants reported using MSUS to examine structures such as bursa/fat pad (89%), ligaments (84%), bone (83%), nerve (81%), muscle (77%), vascular system (69%) and other (including, cysts, features of gout, plantar plate, scar tissue, tumours) (11%). Four participants indicated that they were not trained to image lower limb tissues other than tendon or fascia. For tendon/fascia tissue, the three most commonly imaged structures were the plantar fascia (98%), Achilles tendon (94%) and tibialis posterior tendon (91%).

### Training and use of MSUS

99% of participants (*n* = 135) reported that they routinely scan the foot in addition to the ankle (88%), knee (12%), lower limb (4%) and hip (2%). A small proportion (3%) said that they scanned the upper limb, however those reporting this said that they were working in an extended scope role and had completed an appropriate qualification to do so. In most cases, podiatrists performed MSUS examinations regularly as part of their caseload: 1–10% of their time (30%), followed by 21–30% of their time (18%), 11–20% of their time (16%), 31–40% of their time (10%). 81% of participants (*n* = 225) reported having attended events which provided informal training in MSUS. Informal training was categorised as in-service training, mentoring, supervision or professional activities. 62% reported attending formal training events in MSUS categorised as a structured teaching programme, delivered by trained professionals for an educational institution such as a university or teaching college. Most podiatrists reported using MSUS for 1–5 years (77%) with fewer podiatrists using MSUS for 6–10 years (13%), 11–20 years (9%) and one podiatrist reporting they have used it between 21 and 30 years (0.7%).

### Impact of the use of MSUS imaging on podiatry practice and patient care

A range of free text comments were received from the participants in response to the question relating to their thoughts on the impact of using MSUS imaging in their practice (*n* = 109) and on their perceptions of how the use of MSUS has influenced their approaches to management of their patients’ musculoskeletal foot and ankle problems (*n* = 108). Thematic analysis of the free text comments generated four themes: (i) diagnosis, (ii) delivery and access of care, (iii) patient education and engagement, and (iv) patient empowerment.

(i) *Diagnosis*

Recurring comments within this theme revolve around the ability to use MSUS imaging to define foot pathology that was undetectable through clinical examination alone.

*“[ultrasound] Takes out the guess work – It can be more definitive, saves time in requesting USI [ultrasound image] from radiology, improves the patient journey and reduces the number of patient appointments.’*

*‘ … .managed to avoid performing an unnecessary ‘search’ for a foreign body [FB] as the FB the patient thought they had turned out to be an accessory ossicle and [was] therefore referred to the surgeon.’*

*‘It helps to narrow down a diagnosis and get the correct management plan sooner. Greater confidence in diagnosis especially and tracking of progress to a lesser extent’.*

*‘Biggest game changer to my practice and also to our MSK [musculoskeletal] foot and ankle service.’*

*‘Patients always comment that it is ‘amazing’ that they can see ‘inside their foot’ and can’t believe that they can have this opportunity at a private community clinic.’*

*‘US [ultrasound] becomes our eyes within the foot. I couldn’t imagine my practice without it now’*

(i) *Delivery and access of care*

This theme formed the largest section of comments that related to patients having improved access to treatments, ‘informed’ referrals, reduced waiting time for appointments, targeted injection techniques, improved patient journeys, well-developed management plan, and appropriate allocation of resources.

*‘For me it adds new metrics for clinical audit - pre- post- measures and aids decision making to refer for other imaging etc.’*

*‘Reduction in waiting times for imaging as can be completed within the clinic. Used for guided injections increasing patient confidence.’*

*‘Improved patient journey, improved waiting times for ultrasound, results now at time of scan and any care plan changes at time of scan, improved access to guided injections, reduced referrals to orthopaedics and radiology and magnetic resonance imaging (MRI). Saves our NHS approx. £70,000 per year with podiatry led us service.’*

*‘Helps support your clinical findings. Pts [patients’] tend to be more engaged with rehab [rehabilitation]* etc. *Less time waiting for a referral to radiology. Better patient journey.’*

*‘Huge impact. Not only as a point of care tool but also providing full diagnostics and present options as first contact service.’*

(ii) *Patient education and engagement*

Common remarks within this theme stated the benefit of using MSUS in explaining to their patients what the problem was in their foot/feet. Comments also indicated that this led to improved engagement with treatment plans. Some, however, cautioned of a potential overreliance on the visual image at the detriment of the clinical history and also expressed caution in the use of MSUS over time to determine recovery of symptoms.

*‘Improves patients and clinical understanding of what is causing their pain enabling them to make an informed choice on their treatment options. However, patients do become focused on the result of the imaging not on what they are actually feeling.’*

*‘Since it is non-invasive, patients tend to tolerate it much more than other modalities such as magnetic resonance imaging (MRI), computed tomography (CT), ...etc. Also, it is much easier to explain pathologies to patients since they can see their scans being carried out in real-time, accompanied by dynamic testing. Injuries can also be followed-up, and thus patients can actually see their progress over time. Overall, in my opinion, ultrasound imaging has quite a great positive impact on patients.’*

*‘It is a similar effect to when I started routinely asking about medication history in the 1990s! They made comments inferring that they didn’t know podiatrists knew about that kind of thing. USI [ultrasound imaging] adds a ‘wow’ in the first instance but then offers a window to discuss anatomy and pathology that they can contribute to. It elevates the conversation and engagement between podiatrist and patient.’*

*(iv) Empowering patients.*

Many comments within this theme overlapped with the theme of *patient education and engagement*. Again, all podiatrists’ comments related to a positive effect of the use of MSUS in their practice that emphasised the aspects of patients being able to ‘see’ their foot problem.

*‘I think it [MSUS] adds to the patient empowerment process when they can see the problem.’*

*‘Improves patient comprehension of the pathology by visualizing the problem first-hand and assures patients of the correct diagnosis. Taken together, US [ultrasound] therefore facilitates patient co-operation in following the treatment plan.’*

*‘Patients love seeing what the pathology is and increases their confidence in the diagnosis and treatment. Often patients are satisfied with the confirmation of the diagnosis and are more likely to self-manage.’*

## Discussion

Since Bowen et al. (2008) first highlighted that podiatrists could use MSUS reliably as an additional clinical tool, over the past thirteen years it is evident that the technique is becoming more widely adopted [16]. Findings from our survey support observations of growth in the use of, and training in, MSUS imaging within podiatry in the UK [11] and provide new data on the uptake and use.

Our data provides additional information regarding the context in which MSUS is used by podiatrists working as part of larger multidisciplinary teams or individually depending on their care or access pathways in both the public and private health sectors. Our survey has also revealed novel insights into why podiatrists use MSUS and their thoughts on how this impacts their patient care and practice. Acknowledging the focus of this investigation has been on eliciting podiatrists’ perceptions of their use of USI to benefit patient care. Further research investigating patient perceptions is warranted to evaluate the benefits of MSUS in practice is needed before recommendations could be made.

Of 232 podiatrists who completed our survey and were regular users of MSUS there was good evidence of the uptake. These findings are similar to those reported in the physiotherapy profession by Ellis et al. [10]. Ellis et al. [10] illustrated the wide global reach of ultrasound imaging in physiotherapy, with 60% of respondents being users in Europe, 20% in Australasia and 10% in North America. Within Europe, 47% of UK physiotherapy respondents were users, with higher proportions in the Netherlands (82%) and Spain (69%); where MSUS is already implemented in pre-registration physiotherapy training.

Ellis et al. [10] recruited 1307 physiotherapists to better understand how physiotherapists use MSUS and what barriers prevent its use. Of their respondents, 495 (38%) were users of MSUS. Comparably, findings from our survey indicate that podiatrists use MSUS for diagnosis and delivery/access of care e.g. injection administration and as a diagnostic tool adjunct to clinical assessment to support decision making.

The number of podiatrists responding to our survey was smaller (*N* = 232), however, we specifically recruited only podiatrists who were current users of MSUS to explore the uptake and use of MSUS imaging and the impact they perceive this advanced skill has on their patients and services. Most notably, half of our respondents (*n* = 108) (57%) indicated that there was a consequent high positive impact to their practice due to their ability to use MSUS imaging at the point of care with their patients. Podiatrists reported that the ability to use MSUS imaging to define foot pathology that was undetectable through clinical examination alone enabled them to make better ‘informed’ referrals, creating improved efficiency in the referral pathways. Subsequently patient care pathways and access to care was also reported to be improved.

MSUS used by podiatrists thus has the potential to directly impact the delivery, burden, access of care by reducing time delays and the number of appointments currently required for confirmation of foot and ankle musculoskeletal pathologies [17]. It could be hypothesised that this could reduce financial costings. This has been demonstrated in conditions affecting the shoulder and hip however, this has yet to be determined for the foot and ankle [18]. Woodburn et al. [19] proposed a new paradigm for podiatry care in early rheumatoid arthritis, that there is an argument for a shift towards the use of MSUS imaging being embedded as a key skill for podiatrists working within the field of in musculoskeletal health across both private and public sectors although there needs to be further exploration around efficacy. To achieve this, training standards and competencies for podiatrists using MSUS are essential as well further evidence on cost effectiveness as arguably increased appointment times, to obtain MSUS images, could reduce the number of patients seen per day.

With appropriate governance, there is potential for a new model of podiatric practice to emerge in which a holistic approach using technology to enhance diagnosis and management of musculoskeletal conditions or pathologies also facilitates patient education, engagement and empowerment [20–22]. The findings from our survey indicated common benefits of podiatrists using MSUS in their practice including improved access, reduced waiting times for appointments, improved patient journeys through tighter, more focused management plans. Notably, the use of MSUS imaging by podiatrists, as a means to better inform and educate patients of their diagnosis, is a novel finding that emerged as a key theme in our analyses. Podiatrists responding to open questions in our survey believed that their use of MSUS led to higher levels of empowerment and consequent engagement of their patients with prescribed management plans. Podiatrists also reported positive feedback from their patients that changed their understanding of what the scope of knowledge and practice of podiatrists is. Perceptions surrounding professional status has long been an issue for podiatrists, with many reports of negative connotations towards the podiatry profession due to lack of understanding from other health professionals on the knowledge and skill set that podiatrists possess [22–25]. The embedding of a standard model of practice for the use of MSUS by podiatrists therefore has potential to change the professional landscape, inherently increase the workforce capabilities of the podiatry profession, support better allocation of health resources and overall improve patient care and experiences [21]. To achieve this, collaborative work between professional associations and accreditation registries for sonography internationally are recommended. There is a need for future research in the advocation of agendas related to MSUS in podiatry. This in turn, will further promote the high standards and value the profession upholds in terms of delivering quality, safe and timely care to patients could be replicated in other countries [20].

### Strengths and potential limitations

Our findings are within the limitations of the survey; therefore, some findings may not be generalisable to all countries and their health systems. However, findings do provide some insight around the potential use and benefit of MSUS by podiatrists internationally. We appreciate that further exploration should be undertaken before advocating the widespread use of MSUS into podiatric practice at point of care.

Our survey built on previous surveys of podiatrists and physiotherapists [10, 11]. We used a group of experts derived from four different countries to check face validity until agreement was achieved. The use of quantitative data in combination with qualitative responses to evaluate our aims supported understanding through an inductive approach that expanded on the deductive knowledge of the use of MSUS by podiatrists.

There are some potential limitations of this survey. Firstly, whilst SurveyMonkey was an easily accessible platform, inherent in the use of an internet-based survey is the bias towards respondents who have access to information technology.

Secondly, the survey was conducted in English. We did not have the resources to translate the survey from English to different languages and so this may have affected our international reach. Whilst we are confident that we have reached a large proportion of our target population, there may be some under or over estimation of findings in particular from non-English speaking countries [22].

Thirdly, it is not possible to know how many podiatrists our survey reached, therefore non-response rates cannot be determined and the proportion of the podiatry profession using MSUS imaging cannot be determined. This is a known limitation of the sampling method [26, 27]. This potentially reduces confidence in the results found as the use of MSUS imaging by podiatrists may differ between countries, in particular those not represented within our participants. It was not our intention to find out about the health systems and practices in terms of its global picture although maybe an aspect to consider in the future.

Moreover, it was not possible to track each respondent therefore we are unable to understand why some participants choose to answer some of the questions but not others. It was also not possible to monitor who had been invited in order to work out who might have been missed, which in turn could have underrepresented the collective participant voice. There is a potential for availability heuristic to be present when participants were recalling information about MSUS, this could have impacted how participants responded to the survey by only being able to recall the most recent memorable examples which may not have be representative of a typical day using USI in practice. Therefore, this survey is based on the anecdotal experience of podiatrists and the reports the survey responders have made on cost benefit, improved access, reduced waiting times for appointments, improved patient journeys through tighter and more focused management plans.

Finally, the podiatrists in our survey are obvious champions for the use of MSUS in the foot and ankle and as the observations have not come directly from patients there may be a positive bias to our findings. An essential next step is to explore the thoughts and values of stakeholders to better understand their experiences of MSUS examination used within different health settings.

## Conclusion

Over time, MSUS imaging has diversified to be accessible in rheumatology, sports podiatry, surgery and biomechanics. Our findings confirm those of previous authors in that MSUS imaging is a technique that is being adopted by podiatrists across the globe in both private and public health care sectors as an additional skill to aid diagnosis, assess disease severity and monitor the effect of therapeutic management of foot and ankle pathology. The key benefit that podiatrists reported is improvement in the patient journey, through tighter, focused management plans and reduced waiting times. An additional novel finding was that MSUS provided the capacity for podiatrists to better inform patients of their diagnosis, which they believed led to improved engagement and consequent empowerment of patients in their treatment plans. We propose further investigation of patient experiences and testing of this model that embeds podiatrists’ use of MSUS as a key skill in musculoskeletal foot and ankle services.

## Supplementary Information

**Additional file 1.** Survey Development Process.

**Additional file 2.** Questionnaire.

**Additional file 3.** Flow Diagram: Thematic Analysis.

**Additional file 4.** Checklist for Reporting Results of Internet E-Surveys (CHERRIES).

## Data Availability

The anonymised data that support the findings of this study are available from the corresponding author upon reasonable request.

## Abbreviations

CASE: Consortium of Sonographic Education
CT: Computerized tomography
FB: Foreign Body
MRI: Magnetic resonance imaging
MSK: Musculoskeletal
MSUS: Musculoskeletal ultrasound
NHS: National Health Service
UK: United Kingdom
US: Ultrasound
USI: Ultrasound Imaging
