# Supplementary material for: Exploring the use of musculoskeletal ultrasound imaging by podiatrists: an international survey
Source: J Foot Ankle Res. 2021 May 12;14:39. doi: 10.1186/s13047-021-00478-4 (PMC8114514; doi:10.1186/s13047-021-00478-4)
Supplement: Supplementary file 1 — Additional file 1. Survey Development Process. [file 13047_2021_478_MOESM1_ESM.docx]

**Anaylsis and Results**

Descriptive data (n= 20 questions)

Thematic analysis (n= 2 questions)

**Survey Dissemination Strategy**

Posting on social media pages of special interest groups , author networks, twitter

Virtual advertising repeated monthly for 3 months

Pilot testing survey in Survey Monkey (n=5). 2 responders were part of the collaborative team (LC,CB) and 3 responders were collegues/students at the University of Southampton (EC, MS, HR)

Survey design reviewed and refined

**Survey Draft 6**

Subject Experts Review (n=9). (CD, RE, MC, PM, GGN, HS, LC, AG, CB)

Survey design in Survey Monkey reviewed and revised.

Ordering of questions reviewed and revised

Subject Experts Review (n=9). Iterations made. (CD, RE, MC, PM, GGN, HS, LC, AG, CB)

Culture sensitvity of terms and question content reviewed and revised

Consideration of assumptions, beliefs and values about MSUS/Health Systems

Subject Experts Review (n=9). Iterations made. (CD, RE, MC, PM, GGN, HS, LC, AG, CB)

Culture sensitvity of terms and question content reviewed and revised

Consideration of professional licence and goverance

**Survey Development**

**Agreement and Refinement**

**Final Survey**

**Survey Draft 5**

**Survey Draft 4**

**Survey Draft 3**

Subject Experts Review (n=4). Iterations made (CD, RE, MC, CB)

Consideration of cultural, professional training and health system variations

**Survey Draft 2**

Subject Experts Review (n=4). Iterations made. (CD, RE, MC, CB)

Consideration of podiatric, muscuslokeletal and ultrasound phrases/terminology

**Survey:** Exploring the use of musculoskeletal ultrasound imaging by podiatrists: an international survey

**Survey Development**

Reviewing methods, analysis and findings from Siddle et al (2018) and Ellis et al (2018)

**Survey Draft 1**
