# Supplementary material for: Exploring the use of musculoskeletal ultrasound imaging by podiatrists: an international survey
Source: J Foot Ankle Res. 2021 May 12;14:39. doi: 10.1186/s13047-021-00478-4 (PMC8114514; doi:10.1186/s13047-021-00478-4)
Supplement: Supplementary file 2 — Additional file 2. Questionnaire. [file 13047_2021_478_MOESM2_ESM.pdf]

Participant Information Sheet

Project Title: Exploring the clinical use of ultrasound imaging by podiatrists: An international survey

An Invitation

What is the research about?
Thank you for considering the opportunity to participate in this survey. My name is Professor Catherine Bowen and I lead an international group of researchers and clinicians conducting a study to explore how ultrasound imaging may or may not be used by podiatrists. Our survey includes 25 questions about the use of ultrasound imaging as part of your podiatry practice.

Why have I been asked to participate?
You are reading this participant information form because you have clicked on the link to the survey titled "Exploring the clinical use of ultrasound imaging by podiatrits: An international survey"

What will happen to me if I take part?
Once you finish reading this form and click the 'next' button, the website will offer you a number of questions to complete. By clicking the 'next' button you are consenting to taking part in this survey.

The majority of the survey consists of questions with tick-box answers. For those podiatrists who are not using ultrasound imaging, it is anticipated the survey will take approximately 5 minutes to complete. For those podiatrists who are using ultrasound imaging, it is anticipated the survey will take approximately 10 minutes to complete. On completion of the questions, you will be asked to submit the survey. On doing so, the answers will be submitted to me. The submitted answers will be anonymous, meaning I will not know who they have come from.

Are there any benefits in my taking part?
There may be no direct benefit to you taking part in the survey. However, there may be a benefit to others perhaps, or the study may help improve our current understanding of the area.

On completion of the survey, you will be offered the opportunity to enter a prize draw for an Amazon Gift Card, worth £250.00. To enter all you need to do is enter your email address in the space indicated within the survey. Your email address will not be connected to your survey answers in any way. Your email address is needed so we can contact you if you are the winner of the prize draw. Therefore, there will be no way of knowing what you wrote in the survey.

Are there any risks involved?
There are no anticipated risks in completing this survey.

What data will be collected?
The survey will ask you briefly about your professional details, scope of practice and percieved impact on patient care. Answers to these sections will be either via a tick box selection on the questionnaire or a free text box to type your answers.

Will my participation be confidential?
Your participation and the information we collect about you during the course of the research will be kept strictly confidential. Only members of the research team and responsible members of the University of Southampton may be given access to data about you for monitoring purposes and/or to carry out an audit of the study to ensure that the research is complying with applicable regulations. Individuals from regulatory authorities (people who check that we are carrying out the study correctly) may require access to your data. All of these people have a duty to keep your information, as a research participant, strictly confidential.

Do I have to take part?
No, it is entirely up to you to decide whether or not to take part. If you decide you want to take part, you will need to 'tick' the appropriate sections on the webpage questionnaire to show you have agreed consent to take part.

What happens if I change my mind?
You have the right to change your mind and withdraw at any time without giving a reason and without your participant rights being affected. If you decide to withdraw from the survey the data you have entered will not be recorded.

What will happen to the results of the research?
Your personal details will remain strictly confidential. Research findings made available in any reports or publications will not include information that can directly identify you without your specific consent.

Where can I get more information?
If you have any questions about the survey and wish to talk to a member of the research team, please contact, Dr Charlotte Dando on cd1a19@soton.ac.uk.

What happens if there is a problem?
If you have a concern about any aspect of this study, you should speak to the researchers who will do their best to answer your questions. Please contact, Dr Charlotte Dando on cd1a19@soton.ac.uk.

If you remain unhappy or have a complaint about any aspect of this study, please contact the University of Southampton Research Integrity and Governance Manager (023 8059 5058, rgoinfo@soton.ac.uk).

Thank you for your time

Consent to participate in the survey

* 1. I wish to take part in this study, have read the information sheet on the previous page and have been given adequate time to make this decision. *(Please choose the appropriate option)*

◯ Yes

◯ No

* 2. I am a registered podiatrist (e.g. registered with a national licensing organisation/ registration authority) *(Please choose the appropriate option)*

◯ Yes

◯ No

PROFESSIONAL DETAILS (1)

* 3. I practice as a podiatrist/podiatric surgeon in the following country: *(If you practice in more than one country, choose the country where you spend most of your time)*

- Australia
- Belgium
- Brazil
- Canada
- China
- Denmark
- Egypt
- France
- Finland
- Germany
- Greece
- Hungary
- India
- Ireland
- Italy
- Japan
- Korea
- Malta
- New Zealand
- Norway
- Poland
- Portugal
- South Africa
- Spain
- Sweden
- The Netherlands
- United Arabic Emirates
- United Kingdom
- United States of America
- Other (please specify)

PROFESSIONAL DETAILS (2)

* 4. My work is predominantly: *(Please choose the appropriate options)*

- [ ] Clinical
- [ ] Management
- [ ] Teaching/Education
- [ ] Research
- [ ] Other (please specify)

* 5. I work in the following areas: *(Please choose one or more of the following options)*

- [ ] Public hospital/clinic
- [ ] Private practice
- [ ] Private hospital
- [ ] Private organisation
- [ ] Sports team or sports institute
- [ ] University
- [ ] Research facility
- [ ] Community (e.g. charities, support groups)
- [ ] In a field unrelated to podiatry (e.g. sales, marketing etc.)
- [ ] Other

* 6. I work in the following areas: *(Please choose one or more of the following options)*

- [ ] Musculoskeletal/biomechanics
- [ ] Neurology
- [ ] Vascular
- [ ] Rheumatology
- [ ] High Risk Foot/ Diabetes
- [ ] Podiatric Surgery
- [ ] Nail Surgery
- [ ] Dermatology
- [ ] Sports Medicine
- [ ] Paediatrics
- [ ] General podiatry (corns and callus debridement included)
- [ ] Other (please specify)

PROFESSIONAL DETAILS (3)

* 7. I have the following work experience, as a podiatrist, in years: *(Please choose the appropriate option)*

- ○ 0-5 years
- ○ 6-10 years
- ○ 11-15 years
- ○ 16 - 20 years
- ○ 25 years plus

* 8. In which country did you attain your entry level podiatry qualification? *(Please choose the appropriate option)*

- ◯ Australia
- ◯ Belgium
- ◯ Brazil
- ◯ Canada
- ◯ China
- ◯ Denmark
- ◯ Egypt
- ◯ Finland
- ◯ France
- ◯ Germany
- ◯ Greece
- ◯ Hungary
- ◯ India
- ◯ Ireland
- ◯ Italy
- ◯ Japan
- ◯ Korea
- ◯ Malta
- ◯ New Zealand
- ◯ Norway
- ◯ Poland
- ◯ Portugal
- ◯ South Africa
- ◯ Spain
- ◯ Sweden
- ◯ The Netherlands
- ◯ United Arabic Emirates
- ◯ United Kingdom
- ◯ United States of America
- ◯ Other (please specify below)

Other (please specify)

* 9. Have you attended any INFORMAL training in ultrasound/sonography? *(Please choose the appropriate option)*

Informal being work-based continuing professional development (CPD) (e.g. inservice training, staff/student supervision, etc.) or professional activities CPD (e.g. non-paid branch meeting)

- ○ Yes
- ○ No

10. Have you attended any FORMAL training in ultrasound/sonography? (Please choose the appropriate option)

Formal being a structured teaching programme delivered by trained professionals from an educational institute (e.g. university, teaching college)

- ○ Yes
- ○ No

* 11. Have you been awarded a postgraduate qualification? *(Please choose the appropriate option. If you hold more than 1 qualification, please check the option of your highest qualification)*

- ○ No, I do not hold a postgraduate qualification
- ○ Postgraduate certificate
- ○ Postgraduate diploma
- ○ Masters degree (i.e Masters of Philosophy (MPhil), Masters of Health Science (MHSc), Masters of Science (MSc), Masters of Health Practice (MHPrac), etc.)
- ○ Graduate entry doctoral degree (i.e. Doctor of Podiatry, DPT, etc.)
- ○ Doctoral degree (i.e. Doctor of Philosophy (PhD), Doctor of Health Science (DHSc), etc.)
- ○ Doctor of Podiatric Medicine (DPM)
- ○ Other

ULTRASOUND IMAGING PRACTICE (1)

There are different uses of ultrasound imaging (USI). The following questions refer to these different uses.

12. Which types of ultrasound imaging are you aware of? *(Please choose one or more of the following options)*

- ☐ Diagnostic ultrasound imaging (e.g. diagnosis of musculoskeletal pathology)
- ☐ Interventional ultrasound imaging (e.g. guiding percutaneous procedures; acupuncture)
- ☐ Rehabilitative ultrasound imaging (e.g. measurement of muscle parameters, muscle recruitment)
- ☐ Research ultrasound imaging
- ☐ Other (please specify)

* 13. Do you, in your licensing jurisdiction (i.e. country, province, state) have a formal accreditation to use ultrasound imaging? *(Please choose the appropriate option).*

- Yes
- No
- I don't know

ULTRASOUND IMAGING PRACTICE (2)

* 14. Do you personally perform a ultrasound examination? *(This does **NOT** include referring patients to another health professional for an ultrasound scan, but perform ultrasound imaging yourself. Please choose the appropriate option)*

- Yes
- No

ULTRASOUND IMAGING PRACTICE (3)

* 15. How many years have you been using ultrasound imaging (USI)? *(Please choose the appropriate option)*

- 1-5 years
- 6-10 years
- 11-20 years
- 21-30 years
- 31-40 years
- 41-50 years
- 50+ years

* 16. I use ultrasound imaging (USI)... *(Please choose one or more of the following options)*

- [ ] To conduct research to examine new USI techniques
- [ ] For training other podiatrists/podiatric surgeons how to use USI
- [ ] For training other clinicians (e.g. physiotherapists, GP's etc.) how to use USI
- [ ] For measuring cross-sectional area (CSA) and/or volume of soft tissues (e.g. muscles, tendons, nerves etc.)
- [ ] For measuring linear soft tissue (e.g. muscles, tendons, nerves etc.) thickness and/or width
- [ ] For evaluating muscle structure (e.g. shape, pennation angle, muscle fascicle length, fatty infiltration etc.)
- [ ] For monitoring outcome of treatment (e.g. from comparing a baseline parameter to the same parameter at follow-up)
- [ ] As an indicator for doppler activity
- [ ] As a biofeedback tool
- [ ] For the assessment of soft tissue trauma and monitor healing
- [ ] To assist in making a diagnosis of injury and/or pathology
- [ ] For guiding percutaneous procedures (e.g. acupuncture, dry needling, needle guidance etc.)
- [ ] Other (please specify)

* 17. Which regions of the body do you routinely scan? *(Please choose one or more of the following options)*

- [ ] Hip
- [ ] Knee
- [ ] Ankle
- [ ] Foot
- [ ] Other (please specify below)

Other (please specify)

* 18. What percentage of your caseload are you performing a diagnostic ultrasound examination on each month? *(Please choose the appropriate option)*

- ◯ Not applicable, I do not use USI on patients
- ◯ 1-10%
- ◯ 11-20%
- ◯ 21-30%
- ◯ 31-40%
- ◯ 41-50%
- ◯ 51-60%
- ◯ 61-70%
- ◯ 71-80%
- ◯ 81-90%
- ◯ 91-100%

ULTRASOUND IMAGING PRACTICE (4)

* 19. Which tendons/fascia do you examine using ultrasound imaging (USI)? *(Please choose one or more of the following options)*

- ☐ Extensors (hallucis longus and digitorum longus)
- ☐ Peroneal (longus and brevis)
- ☐ Flexors (hallucis longus and digitorum longus)
- ☐ Tibialis anterior
- ☐ Tibialis posterior
- ☐ Achilles
- ☐ Plantar Fascia
- ☐ Other (please specify)

* 20. What other structures do you examine using ultrasound imaging (USI)? *(Please choose one or more of the following options)*

- [ ] No, I have not been trained to image other tissues
- [ ] Bone
- [ ] Ligament
- [ ] Nerve
- [ ] Muscle
- [ ] bursa/fat pad
- [ ] Vascular system (e.g. blood vessels)
- [ ] Other (please specify)

CLINICAL IMPACT

21. What do you think, if any, is the impact to using ultrasound imaging in your practice?

1 (no impact)				5 (some impact)					10 (high impact)
○	○	○	○	○	○	○	○	○	○

Please add any additional comments

22. In your opinion, what is the impact on your patients to using ultrasound imaging in practice?

Survey Completion

Thank you for taking part in this survey. Your time and answers are greatly appreciated.

If you would like to enter the draw to win the survey prize (Amazon.com Gift Card, valued at £250 GBP), then please enter your email address below.

23. Please enter your email address to go into the prize draw:

Please note that by providing your email address for the prize draw you may identify yourself to the researchers. However the data will not be able to be matched to you personally and your participation will remain anonymous.
