# Supplementary material for: Exploring the use of musculoskeletal ultrasound imaging by podiatrists: an international survey
Source: J Foot Ankle Res. 2021 May 12;14:39. doi: 10.1186/s13047-021-00478-4 (PMC8114514; doi:10.1186/s13047-021-00478-4)
Supplement: Supplementary file 3 — Additional file 3. Flow Diagram: Thematic Analysis. [file 13047_2021_478_MOESM3_ESM.docx]

**Final Themes were: Diagnosis, Delivery and access of care, Patient education and engagement, Patient Empowerment**

Online meeting to share and refine themes and codes with collaborative feedback (CD, CB)

Discussion, justification and agreement on themes and the codes generated

(CD, RE, MC, PM, GGN, HS, LC, AG, CB)

Shared themes and codes with collaborative team (CD, RE, MC, PM, GGN, HS, LC, AG, CB)

Discussion and thoughts generated as a collective team. Feedback documented.

(CD, RE, MC, PM, GGN, HS, LC, AG, CB)

Online meeting to share and refine themes and codes (CD, CB)

Discussion, justification and agreement on themes and codes generated

Additional key themes added were: **Patient Perceptions, Delivery and Access to Care, Patient Adherence, Education**

Independent refinement of themes and codes

Independent refinement of themes and codes

Online meeting to share themes and codes (CD, CB)

Discussion, justification and agreement on themes and codes generated

Key Themes were: **Diagnosis, Management Planning, Profession, Perception**

Constructed themes and codes

**Reviewer 2 (CB)**

Independently reviewed data

**Reviewer 1 (CD)**

Independently reviewed data

**Survey:** Exploring the use of musculoskeletal ultrasound imaging by podiatrists: an international survey
